# Health-related quality of life among persons with initial mild, moderate, and severe or critical COVID-19 at 1 and 12 months after infection: a prospective cohort study

**DOI:** 10.1186/s12916-022-02615-7

**Published:** 2022-11-02

**Authors:** Anouk Verveen, Elke Wynberg, Hugo D. G. van Willigen, Udi Davidovich, Anja Lok, Eric P. Moll van Charante, Menno D. de Jong, Godelieve de Bree, Maria Prins, Hans Knoop, Pythia T. Nieuwkerk, the RECoVERED Study Group, Ivette Agard, Jane Ayal, Floor Cavdar, Marianne Craanen, Annemarieke Deuring, Annelies van Dijk, Ertan Ersan, Laura del Grande, Joost Hartman, Nelleke Koedoot, Tjalling Leenstra, Romy Lebbink, Dominique Loomans, Agata Makowska, Tom du Maine, Ilja de Man, Amy Matser, Lizenka van der Meij, Marleen van Polanen, Maria Oud, Clark Reid, Leeann Storey, Marc van Wijk, Joost van den Aardweg, Joyce van Assem, Marijne van Beek, Thyra Blankert, Maartje Dijkstra, Orlane Figaroa, Leah Frenkel, Marit van Gils, Jelle van Haga, Xiaochuan Alvin Han, Agnes Harskamp-Holwerda, Mette Hazenberg, Soemeja Hidad, Nina de Jong, Neeltje Kootstra, Lara Kuijt, Colin Russell, Karlijn van der Straten, Annelou van der Veen, Bas Verkaik, Gerben-Rienk Visser

**Affiliations:** 1 Department of Medical Psychology (J3-2019-1), Amsterdam UMC location AMC University of Amsterdam, Meibergdreef 9, 1105 AZ Amsterdam, the Netherlands; 2 Amsterdam Public Health, Amsterdam, The Netherlands; 3 Department of Infectious Diseases, Public Health Service of Amsterdam, Amsterdam, the Netherlands; 4 Department of Infectious Diseases, Amsterdam UMC location University of Amsterdam, Meibergdreef 9, Amsterdam, the Netherlands; 5 Amsterdam Institute for Infection and Immunity, Infectious Diseases, Amsterdam, the Netherlands; 6 Department of Medical Microbiology & Infection Prevention, Amsterdam UMC location University of Amsterdam, Meibergdreef 9, Amsterdam, the Netherlands; 7 Department of Social Psychology, University of Amsterdam, Amsterdam, the Netherlands; 8 Department of Psychiatry, Amsterdam UMC location University of Amsterdam, Meibergdreef 9, Amsterdam, the Netherlands; 9 Center for Urban Mental Health, University of Amsterdam, Amsterdam, the Netherlands; 10 Department of Public & Occupational Health, Amsterdam UMC location University of Amsterdam, Meibergdreef 9, Amsterdam, the Netherlands; 11 Department of General Practice, Amsterdam UMC location University of Amsterdam, Meibergdreef 9, Amsterdam, the Netherlands

**Keywords:** SARS-CoV-2, COVID-19, Quality of life, Health-related quality of life

## Abstract

**Background:**

Currently, there is limited evidence about the long-term impact on physical, social and emotional functioning, i.e. health-related quality of life (HRQL) after mild or moderate COVID-19 not requiring hospitalization. We compared HRQL among persons with initial mild, moderate or severe/critical COVID-19 at 1 and 12 months following illness onset with Dutch population norms and investigated the impact of restrictive public health control measures on HRQL.

**Methods:**

RECoVERED, a prospective cohort study in Amsterdam, the Netherlands, enrolled adult participants after confirmed SARS-CoV-2 diagnosis. HRQL was assessed with the Medical Outcomes Study Short Form 36-item health survey (SF-36). SF-36 scores were converted to standard scores based on an age- and sex-matched representative reference sample of the Dutch population. Differences in HRQL over time were compared among persons with initial mild, moderate or severe/critical COVID-19 using mixed linear models adjusted for potential confounders.

**Results:**

By December 2021, 349 persons were enrolled of whom 269 completed at least one SF-36 form (77%). One month after illness onset, HRQL was significantly below population norms on all SF-36 domains except general health and bodily pain among persons with mild COVID-19. After 12 months, persons with mild COVID-19 had HRQL within population norms, whereas persons with moderate or severe/critical COVID-19 had HRQL below population norms on more than half of the SF-36 domains. Dutch-origin participants had significantly better HRQL than participants with a migration background. Participants with three or more COVID-19 high-risk comorbidities had worse HRQL than part participants with fewer comorbidities. Participants who completed the SF-36 when restrictive public health control measures applied reported less limitations in social and physical functioning and less impaired mental health than participants who completed the SF-36 when no restrictive measures applied.

**Conclusions:**

Twelve months after illness onset, persons with initial mild COVID-19 had HRQL within population norms, whereas persons with initial moderate or severe/critical COVID-19 still had impaired HRQL. Having a migration background and a higher number of COVID-19 high-risk comorbidities were associated with worse HRQL. Interestingly, HRQL was less impaired during periods when restrictive public health control measures were in place compared to periods without.

**Supplementary Information:**

The online version contains supplementary material available at 10.1186/s12916-022-02615-7.

## Background

With the COVID-19 pandemic having been ongoing for more than 2 years, increasing attention is being paid to the long-term impacts on physical, social and emotional functioning, i.e. the health-related quality of life (HRQL), following SARS-CoV-2 infection. Although many COVID-19 patients return to their pre-COVID-19 state of health after the acute phase of infection [1], appreciable numbers of persons report ongoing post-infection sequelae [2].

Previous studies have shown significantly impaired HRQL within the first months after illness onset [3–6]. To date, only a few studies have investigated the long-term HRQL of COVID-19 patients with follow-up periods of 12 months or beyond, showing mixed results with some studies reporting that patients still have impaired HRQL after 1 year [7–10], and others reporting most patients to have good physical and functional recovery [1, 11]. As these studies were almost exclusively conducted among previously hospitalized patients, an important gap in our knowledge remains since the majority of SARS-CoV-2 infections are asymptomatic or mildly symptomatic not requiring hospitalization, and it is well established that long-term post-infection sequelae is not restricted to individuals who initially had severe or critical COVID-19 requiring hospitalization [12].

Restrictive public health control measures to curb the spread of SARS-CoV-2 may also have a negative impact on the HRQL of COVID-19 patients. For example, being required to stay in isolation whilst recovering from COVID-19 can be socially isolating and impact mental health [13], and may continue to have a negative impact after recovery. In addition, general public health measures beyond the isolation period, such as national restrictions on social contacts, travel, work and leisure, may also negatively impact HRQL, irrespective of previous SARS-CoV-2 infection.

The objectives of the present study are to compare HRQL among persons with initial mild, moderate or severe/critical COVID-19 at 1 and 12 months following illness onset using Dutch age- and sex-matched population HRQL norms, to investigate sociodemographic and clinical factors associated with impaired HRQL, and to investigate the impact of restrictive public health control measures on HRQL.

## Methods

### Study design and participants

The RECoVERED study is a cohort of individuals with laboratory-confirmed SARS-CoV-2 infection in the municipal region of Amsterdam, the Netherlands. Enrolment began on 11 May 2020 [14]. Non-hospitalized participants were identified from notification data at the Public Health Service of Amsterdam and enrolled within 7 days of diagnosis. Prospectively enrolled hospitalized participants were identified from admissions to the COVID-19 wards of the Amsterdam University Medical Centers (AUMC). Up to 30 June 2020, a number of hospitalized patients were included retrospectively within 3 months following SARS-CoV-2 infection.

Eligibility criteria included laboratory confirmation of SARS-CoV-2 infection by reverse transcriptase polymerase chain reaction (RT-PCR), age 16–85 years, residing in the Amsterdam region, and adequate understanding of Dutch or English. Excluded were nursing home residents and individuals with mental disorders likely to interfere with adherence to study procedures. For the present analyses, we included all participants with at least 1 month of follow-up and at least one completed HRQL questionnaire on 1 December 2021.

### Study procedures

Study visits at enrolment (D0) and day 7 (D7) of follow-up took place at the participant’s home or on the hospital ward whilst subsequent visits took place at one of two study sites. Past medical history and sociodemographic data were collected during the first month of follow-up by participant interview and/or medical records. Physical measurements (heart rate, respiratory rate [RR], oxygen saturation [SpO_2_]), were measured at D0 and D7, or retrieved from hospital records for retrospectively enrolled participants.

### Definitions

Illness onset was defined as the first day on which COVID-19 symptoms were experienced for symptomatic patients or date of COVID-19 diagnosis for asymptomatic patients.

Clinical severity groups were defined based on WHO COVID-19 severity criteria 16: mild disease as having a RR <20/min and SpO_2_>94% on room air at both D0 and D7; moderate disease as having a RR 20–30/min and/or SpO_2_ 90–94% or receiving oxygen therapy at D0 or D7; severe disease as having a RR>30/min and/or SpO_2_<90% or receiving oxygen therapy at D0 or D7; critical disease as ICU admission due to COVID-19 at any point.

High-risk comorbidities were those identified by the WHO as being associated with severe COVID-19 [15] and include the following: cardiovascular disease (including hypertension), diabetes mellitus, chronic pulmonary disease (excluding asthma), cancer, immunosuppression (excluding HIV, including previous organ transplantation), previous psychiatric illness, renal disease, liver disease and dementia. BMI was coded in kg/m^2^ as <25, underweight or normal weight; 25–30, overweight; or >30, obese. Migration background was categorized as Dutch and non-Dutch origin based on the country of birth of the participant and their parents [16, 17]. Participants of non-Dutch background were further classified as originating from a OECD high-income (HIC) or low-/middle-income country (LMIC) [18]. Highest educational level was categorized as low/medium (none, primary/secondary school; vocational training) or high (university-level).

### Measurement of HRQL

At months 1 and 12 of follow-up, participants completed the Medical Outcomes Study Short Form 36-item health survey (SF-36) [19]. The SF-36 comprises 36 items that address the following 8 dimensions reflecting the respondent’s HRQL: ability to perform usual and vigorous activities (physical functioning), ability to participate in social and occupational activities (social functioning, physical role functioning, and emotional role functioning), mood (mental health dimension), amount of energy and pain (vitality/fatigue and pain dimensions) and perceived current health (general health perceptions). Each dimension is scored from 0 to 100, with higher scores indicating better HRQL.

### Restrictive public health measures

We defined the progression of restrictive measures that was applied in the Netherlands between 10 June 2020, when the first HRQL questionnaire was to be completed, and 1 December 2021, i.e. the date of closure of our database for the present analysis, based on time periods during which specific public health measures were implemented. We distinguished the following restrictive measures: evening curfew (23 January 2021 to 28 April 2021) and closure of bars, cafes and restaurants (14 October 2020 to 5 June 2021), primary schools, including day care (14 December 2020 to 8 February 2021), secondary and vocational education (14 December 2020 to 1 March 2021), higher education (16 December to 25 April 2021), non-essential shops (15 December 2020 to 28 April 2021), entertainment venues (4 November 2020 to 18 May 2021) and gyms/sport centres (15 December 2020 to 18 May 2021) [20]. Due to the overlap in restrictive measures, we divided them in two groups based on time period and scope. We combined closure of bars, cafes, restaurants, entertainment venues and gym/sport centres and further refer to them as restrictions in leisure activities. We combined closure of educational institutions, non-essential shops and evening curfew and further refer to them as restrictions in non-leisure activities.

### Statistical analysis

Characteristics of participants at enrolment were compared between clinical severity groups, using chi-squared tests or analysis of variance, where appropriate.

Participants’ SF-36 scores were compared with published age- and sex-matched Dutch reference adult population norms [21]. The 8 dimensions of the SF-36 scores were converted to standard scores on the basis of the scores of an age- and sex-matched representative reference sample of the adult Dutch population. Standard scores were calculated by dividing the difference between the participants’ SF-36 score and the mean score of the matched reference population by the standard deviations (SDs) of the reference population, as described previously [22]. A standard score thus indicates how many SDs the observed SF-36 score falls below or above the score of the reference population. Consequently, scores of the reference population are set at 0. Because it is similar to the effect size calculation, a mean standard score of 0.20 is considered to indicate a small deviation from the reference population [22], and mean standard scores of 0.50 and 0.80 are considered to indicate moderate and large deviations from the reference population, respectively [23].

We compared HRQL among persons with initially mild, moderate or severe/critical COVID-19 at 1 and 12 months following illness onset and sociodemographic and clinical factors at illness onset up to 1 month associated with more impaired HRQL using mixed linear models. Standard scores relative to the Dutch population for the eight SF-36 dimensions were the dependent variables. COVID-19 severity group and time since illness onset (month 1, month 12) were included as fixed effects. Correlation between repeated measurements within participants was taken into account by including a random intercept. Mixed linear models are maximum likelihood estimation-based techniques which can handle the presence of missing data and still provide valid results without the need for imputation of data under the missing at random assumption.

We considered the following characteristics for inclusion as fixed covariates: BMI category (normal weight, overweight, obese), number of COVID-19 high-risk comorbidities (<3, ≥3), migration background (Dutch-origin, originating from a HIC, originating from a LMIC), highest completed educational level (low/medium, high). We initially looked at the impact on HRQL of having 0, 1, 2 or ≥ 3 comorbidities. If there was a significant impact of number of comorbidities on HRQL, the difference was between the group of participants with ≥ 3 comorbidities versus those with <3 comorbidities. We therefore decided to proceed using the variable with only 2 categories, i.e. <3 and ≥3. We generated a multivariate model for each of the SF-36 dimensions using a backwards selection approach until all sociodemographic and baseline clinical characteristics in the final model had *p*-values <0.05. COVID-19 severity group and time since illness onset were kept in all models irrespective of statistical significance. If SF36-36 dimensions were significantly associated with the number of comorbidities in the multivariate model, we subsequently entered the specific comorbidities in the model, after removing the variable number of comorbidities, and investigated how much of the variance in the particular SF-36 dimension was explained by each comorbidity.

For the COVID-19 severity groups at month 1 and month 12, we checked whether the 95% confidence intervals of the estimated mean standard scores of the eight SF-36 dimensions excluded the value zero, which would indicate that HRQL is significantly different from the age- and sex-matched population norms.

We verified if at least one of the restrictions in leisure activities applied (yes/no) and/or at least one of the restrictions in non-leisure activities applied (yes/no) on the date that a participant completed the SF-36. The restrictive measures were added to the final multivariate mixed linear model for each of the eight SF-36 dimensions as a time-dependent covariate to examine impact on HRQL.

As we had a priori determined that the variables initial COVID-19 severity and time (month 1, month 12) should be kept in all models irrespective of statistical significance, another 6 variables were investigated as potential determinants, of which 3 variables had 3 categories, so that a total of 11 parameters had to be estimated. Consequently, we would need to include at least 220 participants to satisfy the rule of thumb to have at least 10 to 20 participants per predictor variable for a continuous outcome measure. Statistical analysis was conducted using SPSS version 28 (IBM Corp, Armonk, NY). Two-sided *p*-values <0.05 were considered to indicate statistical significance.

## Results

By 1 December 2021, a total of 269 (77%) out of 349 participants enrolled in the cohort had completed at least one HRQL questionnaire. A total of 111 participants completed a questionnaire at both month 1 and month 12, 101 only at month 1 and 57 only at month 12. Of note, not all participants had reached the month 12 time point at closure of the database for the present analyses. Characteristics at enrolment are shown in Table 1. Participants who did not complete questionnaires more often originated from a LMIC, had a lower educational level, more often had severe/critical COVID-19 and had more often been hospitalized (See Additional file 1: Table S1).

**Table 1:** Sociodemographic, clinical (baseline and COVID-19-related) characteristics of RECoVERED study participants

	Mild	Moderate	Severe/critical	***P***-value
	*N*=82	*N*=122	*N*=65	
Sex				0.18
Male	38 (46%)	67 (55%)	40 (62%)	
Female	44 (54%)	55 (45%)	25 (38%)	
Age (years), mean (SD)	43 (17)	48 (15)	57 (12)	<0.001
BMI category				<0.001
Normal weight	48 (59%)	51 (42%)	14 (22%)	
Overweight	21 (26%)	42 (34%)	29 (47%)	
Obese	12 (15%)	29 (24%)	16 (31%)	
Migration background				<0.001
Dutch origin	61 (75%)	73 (61%)	35 (55%)	
OECD high income	11 (14%)	20 (17%)	4 (6%)	
OECD low/middle income	9 (11%)	27 (22%)	35 (39%)	
Highest level of education				<0.001
None, primary, secondary or vocational training	14 (17%)	51 (42%)	35 (55%)	
University education	67 (83%)	70 (58%)	29 (45%)	
Number of COVID-19 high-risk comorbidities				0.032
<3	80 (98%)	113 (93%)	56 (86%)	
3 or more	2 (2%)	9 (7%)	9 (14%)	
COVID-19 high-risk comorbidities				
Cardiovascular	11 (13.4%)	31 (25.4%)	30 (46.9%)	0.001
Diabetes mellitus	3 (3.7%)	10 (8.2%)	15 (23.4%)	0.001
Chronic respiratory disease	1 (1.2%)	8 (6.6%)	10 (15.6%)	0.003
Cancer	5 (6.1%)	7 (5.7%)	4 (6.3%)	0.99
Immunodeficiency	0	3 (2.5%)	2 (3.1%)	0.31
Previous psychiatric illness	5 (6.1%)	7 (5.7%)	3 (4.7%)	0.93
Other^a^	11 (13.6%)	33 (27.3%	16 (25.0%)	0.064
Hospital admission	3 (4%)	53 (43%)	61 (94%)	<0.001
ICU admission	0	0	32 (49%)	<0.001

Continuous variables presented as mean (standard deviation) and compared using analysis of variance; categorical and binary variables presented as *n*(%) and compared using the Pearson *χ*^2^ test (or Fisher exact test if *n*<5)

Clinical severity groups defined as mild as having a RR<20/min and SpO_2_ on room air >94% at both days 0 and 7; moderate disease as having a RR 20–30/min, SpO_2_ 90–94% and/or receiving oxygen therapy at day 0 or 7; severe disease as having a RR>30/min or SpO_2_ < 90% at day 0 or 7; critical disease as requiring ICU admission

COVID-related comorbidities are based on WHO Clinical Management Guidelines and include: cardiovascular disease (including hypertension), chronic pulmonary disease (excluding asthma), renal disease, liver disease, cancer, immunosuppression (excluding HIV, including previous organ transplantation), previous psychiatric illness and dementia

*BMI* body mass index, *OECD* Organisation for Economic Co-operation and Development, *HIC* high-income country, *LMIC* low- or middle-income country, *ICU* intensive care unit

^a^Other includes renal disease, liver disease and dementia

### Impact on HRQL of initial COVID-19 severity

Table 2 shows the impact on HRQL of initial COVID-19 severity, time since illness onset and sociodemographic- and clinical characteristics according to the final multivariate mixed linear models adjusted for confounders. All final models included clinical severity and time since illness onset, as predetermined, and migration background. The model for physical functioning and general health additionally included the number of comorbidities (Table 2). On 7 out of 8 SF-36 dimensions, participants with mild COVID-19 had significantly better HRQL than participants with moderate or severe/critical COVID-19, whereas there was no statistically significant difference in HRQL between participants with moderate and severe/critical COVID-19. The exception was the mental health dimension where participants with mild COVID-19 had significantly better mental health than participants with moderate COVID-19, but there were no significant differences between participants with mild or moderate versus severe/critical COVID-19. HRQL was significantly better at month 12 compared with month 1 on seven out of eight SF-36 dimensions. On the general health perceptions dimension, month 1 scores were not significantly different from month 12 scores.

**Table 2:** Impact on HRQL of initial COVID-19 severity, time since enrolment and sociodemographic- and baseline clinical characteristics

	Initial COVID-19 severity Reference = mild		Time Reference = month 1	Migration background Reference = Dutch-origin		Comorbidities Reference = <3 comorbidities
	Moderate	Severe/critical	Month 12	Low/middle-income country	High-income country	≥3 comorbidities
**Physical functioning**	−0.47 (−0.77 to −0.17) * *p* < 0.001	−0.65 (−1.04 to −0.27) * *p* < 0.001	0.87 (0.61 to 1.14) * *p* < 0.001	−0.50 (−0.90 to −0.11) * *p* = 0.013	−0.85 (−1.17 to −0.52) * *p* < 0.001	−0.54 (−1.06 to −0.03) * *p* = 0.039
**Role physical**	−0.38 (−0.64 to −0.13) * *p* = 0.003	−0.52 (−0.84 to −0.10) * *p* = 0.002	1.24 (1.01 to 1.46) * *p* < 0.001	−0.28 (−0.56 to −0.010) * *p* = 0.046	−0.44 (−0.77 to −0.62) * *p* = 0.011	Not in model
**Bodily pain**	−0.48 (−0.73 to −0.23) * *p* < 0.001	−0.71 (−1.03 to −0.39) * *p* < 0.001	0.64 (0.42 to 0.87) * *p* < 0.001	−0.42 (−0.69 to −0.15) * *p* = 0.003	−0.71 (−1.04 to −0.37) * *p* < 0.001	Not in model
**Vitality**	−0.58 (−0.83 to −0.32) * *p* < 0.001	−0.69 (−1.02 to −0.36) * *p* < 0.001	0.91 (0.69 to 1.14) * *p* < 0.001	−0.31 (−0.59 to −0.03) * *p* = 0.030	−0.93 (−1.27 to −0.60) * *p* < 0.001	Not in model
**Social functioning**	−0.49 (−0.82 to −0.16) * *p* = 0.004	−0.53 (−1.38 to −0.27) * *p* < 0.001	1.13 (0.90 to 1.36) * *p* < 0.001	−0.44 (−0.72 to −0.15) * *p* = 0.003	−1.03 (−1.38 to −0.68) * *p* < 0.001	Not in model
**Mental health**	−0.30 (−0.53 to −0.07) * *p* = 0.01	−0.15 (−0.45 to 0.14) *p* = 0.311	0.36 (0.15 to 0.56) * *p* < 0.001	−0.18 (−0.43 to 0.07) *p* = 0.161	−1.09 (−1.40 to −0.79) * *p* < 0.001	Not in model
**Role emotional**	−0.34 (−0.60 to −0.08) * *p* = 0.012	−0.43 (−0.76 to −0.10) * *p* = 0.012	0.53 (0.30 to 0.76) * *p* < 0.001	−0.96 (−1.31 to −0.62) * *p* < 0.001	−0.24 (−0.53 to 0.05) *p* = 0.101	Not in model
**General health**	−0.53 (−0.77 to −0.28) * *p* < 0.001	−0.55 (−0.87 to −0.23) * *p* < 0.001	0.11 (−0.12 to 0.33) *p* = 0.355	−0.10 (−0.37 to 0.17) *p* = 0.457	−0.50 (−0.82 to −0.17) * *p* = 0.003	−0.46 (−0.89 to −0.02) * *p* = 0.042

Parameter estimates are regression coefficients from mixed linear models adjusted for confounders. All models include a random intercept. CI denotes confidence interval. Parameter estimates can be interpreted as effect sizes (Cohens’ *d*) with values of 0.2, 0.5 and 0.8 indicating small, medium and large effects respectively

*p<0.05

Dutch-origin participants had significantly better HRQL than participants with a migration background from either HIC or LMIC countries on the dimensions physical, social and role physical functioning, bodily pain and vitality. Dutch-origin participants and participants from LMIC had significantly better HRQL than participants from HIC on the dimensions general health, role emotional and mental health.

Participants with three or more COVID-19 high-risk comorbidities had significantly worse HRQL on the dimensions physical functioning and general health than participants with less than three COVID-19 high-risk comorbidities. We subsequently looked at the impact of specific comorbidities. Immunosuppression explained the largest percentage of variance in physical functioning, i.e. 4.6% followed by previous psychiatric illness, i.e. 1.9%. Previous psychiatric illness explained the largest percentage of variance in general health, i.e. 3.6%, followed by immunosuppression, i.e. 3.3% and by the category “other” (renal disease, liver disease and dementia), i.e. 2.9%. Remaining comorbidities explained less than 1% of variance and are therefore considered of negligible influence.

### HRQL compared with age- and gender-matched populations norms

**Fig. 1:**
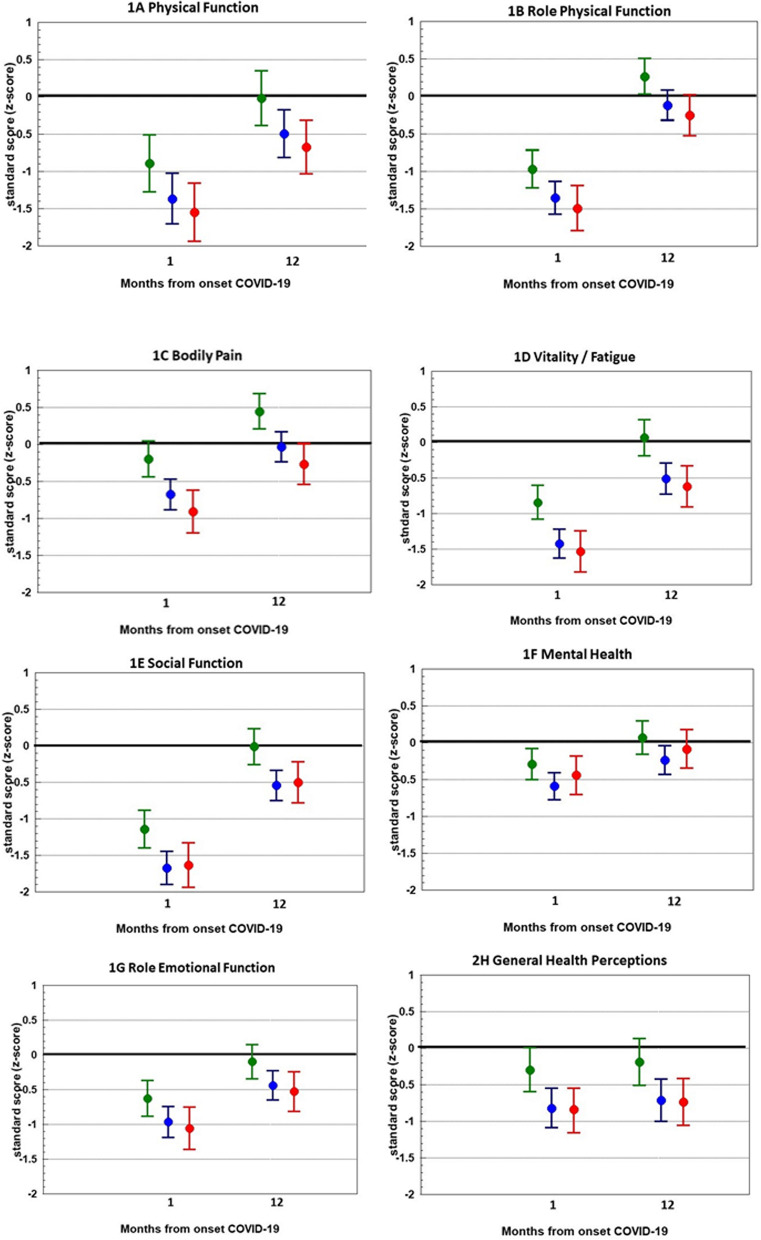
HRQL compared with general population norms. Participants with initial mild COVID-19 are shown in green, initial moderate COVID-19 in blue and initial severe/critical COVID-19 in red. Solid line at zero indicates no difference compared with general population. Values are estimate marginal means with 95% confidence intervals from mixed linear models adjusted for confounders

One month after illness onset, participants had HRQL scores below population norms on seven out of eight SF-36 dimensions (Fig. 1 A–G). These HRQL scores were derived from the final multivariate mixed linear models adjusted for confounders. Deviations from the population norms were highest for social functioning (−1.68, indicating a large effect, for participants with moderate COVID-19) and lowest for bodily pain (−0.20, indicating a small effect, for patients with mild COVID-19). On the general health dimension, participants with mild COVID-19 scored within population norms, whereas participants with moderate or severe/critical COVID-19 had scored below population norms.

**Fig. 2:**
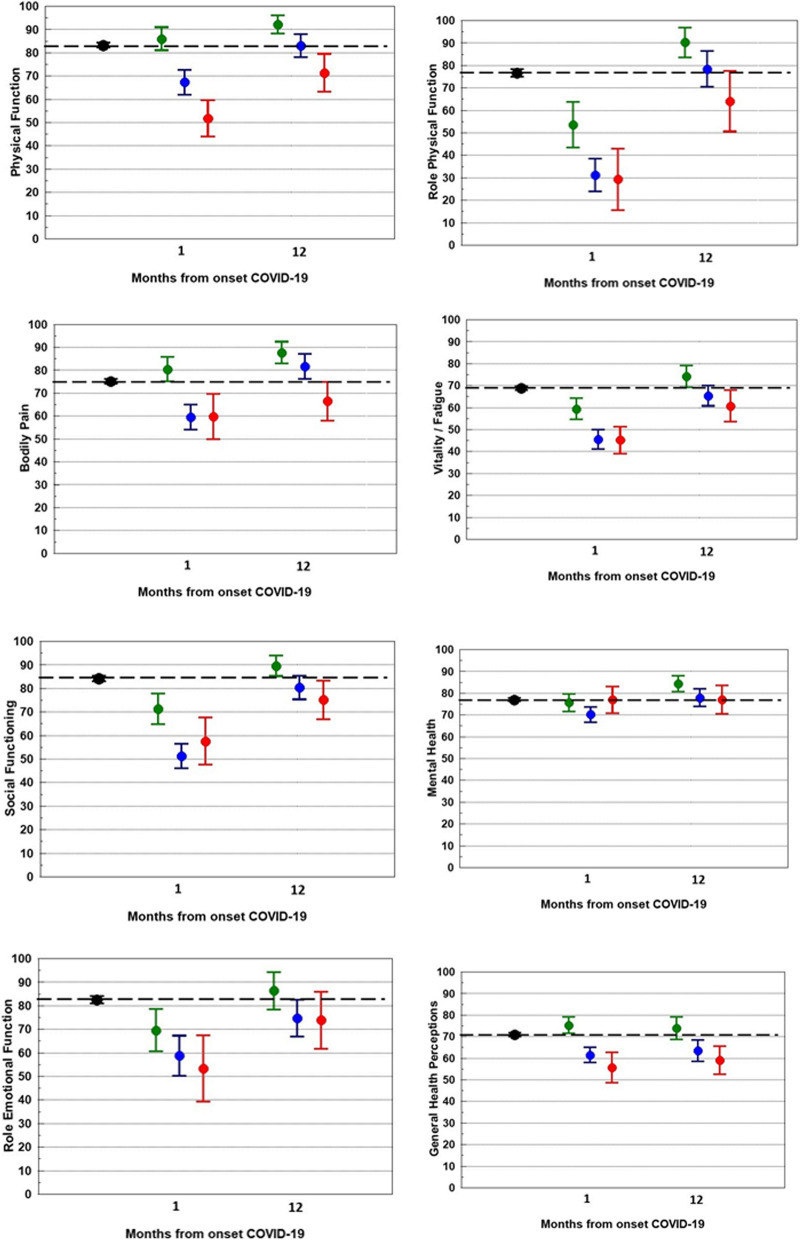
Mean unadjusted HRQL scores compared with mean general population scores. General population is shown in black, participants with initial mild COVID-19 are shown in green, initial moderate COVID-19 in blue and initial severe/critical COVID-19 in red. Dotted line indicates the general population mean. Values are unadjusted mean scores with 95% confidence intervals

After 12 months, HRQL was within population norms on the dimensions bodily pain and role physical in all clinical severity groups. On the dimensions physical functioning, social functioning, vitality, role emotional and general health, participants with initial mild COVID-19 had HRQL within population norms, whereas participants with initial moderate or severe/critical COVID-19 still had HRQL below population norms. On the mental health dimension, only participants with initial moderate COVID-19 had HRQL below population norms. The absolute and unadjusted differences to the population norms are shown in Fig. 2.

### Impact of restrictive public health measures on HRQL

Participants who completed a HRQL questionnaire when restrictions in leisure activities applied reported less impairments in mental health than participants who completed a HRQL questionnaire when no restrictions in leisure activities applied ((mean (se): −0.15 (0.08) versus −0.38 (1.0), *p*=0.039). Participants who completed a HRQL questionnaire when restrictions in non-leisure activities applied reported less limitations in social functioning (mean (se): −0.67 (0.11) versus −0.96 (0.09), *p*=0.042) and in physical functioning (mean (se): −0.68 (0.17) versus −0.99 (0.15), *p*=0.039) than participants who completed a HRQL questionnaire when no restrictions in these activities applied.

## Discussion

In this prospective cohort among participants with mild, moderate and severe/critical initial COVID-19 severity, HRQL was substantially impaired 1 month after illness onset. Twelve months after illness onset, participants with initial mild COVID-19 had HRQL comparable with population norms, whereas participants with initial moderate or severe/critical COVID-19 still had impaired HRQL on five out of eight HRQL dimensions. Interestingly, HRQL was less impaired during periods when restrictive public health control measures applied than in periods when no restrictive measures applied.

To the best of our knowledge, only four previous studies investigating HRQL about 1 year after SARS-CoV-2 infection included non-hospitalized participants. Steinbeis et al. found that HRQL improved over time among patients with higher disease severity, whereas it remained constant among patients in the lower severity categories [24]. Seeβle et al. did not report about HRQL in relation to COVID-19 severity or time since illness onset [10]. Han et al. found that patients with mild COVID-19 not requiring hospitalization who were experiencing persistent symptoms 1 year after COVID-19 had poorer HRQL than patients without persistent symptoms [25]. O’Kelly et al. found that non-hospitalized patients had better mental HRQL and tended to have better physical HRQL than hospitalized patients 1 year after COVID-19 [26].

Our finding that participants with initial mild COVID-19 had HRQL that was, on average, comparable with population norms after 12 months is reassuring, as long-term post-infection sequelae can also occur after mild disease [14]. Participants with initial moderate or severe/critical COVID-19 still had impaired HRQL on more than half of the HRQL dimensions compared with population norms after 12 months. Because there was no assessment of participants’ pre-COVID-19 HRQL, we cannot draw definite conclusions about whether participants had returned to their pre-COVID-19 level of HRQL or if their level of HRQL was still impaired.

At month 12, participants with initial moderate or severe COVID-12 still had impaired HRQL on the dimensions physical, social and role emotional functioning, vitality and general health with moderate to large deviations from general population norms. A previous study comparing these HRQL domains between individuals with and without chronic conditions (arthritis, chronic lung disease, congestive heart failure, diabetes and ischemic heart disease) found decrements of a small to moderate magnitude on these HRQL domains among those with chronic health conditions (26). The time since onset of these chronic conditions was not specified so that participants may have already adapted to living with these chronic conditions. Nevertheless, this implies that the impact of COVID-19 on HRQL after 12 months may still be substantial even if the effect of comorbidity on HRQL is taken into account.

In general, HRQL was less impaired during periods when restrictive public health control measures applied than during periods without restrictive measures. We had expected the opposite as in general populations worldwide, decreases in subjective well-being have been reported as a consequence of restrictive measures [27]. Our result are consistent with social comparison theory according to which it would be expected that participants feel less impaired if other people are also restricted in what they can do. Conversely, if others are no longer restricted in their activities, participants may experience more limitations [28].

Being a migrant from either a LMIC or HIC was associated with having worse HRQL, but this association was more pronounced for HIC. Residing in a foreign country during a global pandemic with an unfamiliar health system when becoming ill can be expected to be particularly challenging. Being more isolated from traditional support networks such as friends and families may also have led to feelings of loneliness and contributed to a negative impact on HRQL. Possibly, the negative impact of COVID-19 on HRQL was less pronounced for participants from LIC because they are more accustomed to cope with adverse circumstances. Another explanation might be that HIC persons could be more accustomed to high quality of care and have higher expectations for such care. It could be that Dutch health care systems potentially fair less well than HIC persons would expect based on their experience back home. This might not be the case for LIC where expectations from the health services are low in comparison to the Netherlands. Clearly, our finding of a more severe impact on HRQL among migrants from HIC compared with LIC needs to be corroborated in other studies.

A point of caution: we compared HRQL with Dutch general population norms in the absence of an uninfected (SARS-CoV-2 negative) control group. These norms were collected before the COVID-19 pandemic. If the COVID-19 pandemic would have a negative impact on the general population’s HRQL, this would render comparing pandemic outcomes with pre-pandemic norms inappropriate. However, the negative impacts of the pandemic on the well-being of general populations worldwide have been reported to be mild and transient [29, 30].

A limitation of this study is that the Dutch population norms were not stratified according to the presence of comorbidities. Of the persons included in the Dutch population norms, 48% had no chronic health condition, 29% had one condition and 23% had more than one condition, but HRQL normative data are not available separately for these groups. Participants with initial moderate or severe/critical COVID-19 more often had medical comorbidities, possibly relating to lower pre-COVID-19 HRQL. Nevertheless, only two of the HRQL domains were affected by the number of comorbidities, i.e. physical functioning and general health perceptions, but these were among the domains that remained below populations norms at month 12.

Participants with initial severe/critical COVID-19, previously hospitalized, a lower educational level and migrants from LMIC were less likely to complete HRQL questionnaires and to be included in the present HRQL study. Given that these factors were also associated with impaired HRQL, this may have resulted in an underestimation of the impact of COVID-19 on HRQL. Another limitation is that persons with mental disorders likely to interfere with adherence to study procedures were not eligible to participate. About 5% of the hospitalized persons were excluded for this reason, and for non-hospitalized persons, this was not registered. Exclusions concerned persons with acute severe mental illness such as delirium and persons with Alzheimer’s disease or intellectual disabilities. Therefore, our results cannot be generalized to persons with delirium, Alzheimer’s disease or intellectual disabilities.

This study also has several strengths. The vast majority of our participants had initial mild or moderate COVID-19 not requiring hospitalization. Currently, this is an understudied group with respect to long-term impacts on HRQL after COVID-19. Our prospective cohort study also follows participants from illness onset, thereby minimizing selection bias resulting from individuals self-referring due to poor HRQL. Another strength is our long follow-up period, allowing us to study truly long-term HRQL impacts of COVID-19.

## Conclusions

Twelve months after illness onset, participants with initial mild COVID-19 had, on average, HRQL comparable with population norms, whereas participants with initial moderate or severe/critical COVID-19 still had impaired HRQL on more than half of the HRQL dimensions. Being a migrant from a HIC was associated with more impaired HRQL. HRQL was less impaired during periods when restrictive public health measures applied than in periods when no restrictions applied.

## Supplementary Information

**Additional file 1: Table S1.** Comparison of demographic and clinical characteristics of RECoVERED study participants who did and did not complete at least 1 HRQL questionnaire.

## Data Availability

Taking legal restrictions into account, the data collected for this study are available on request via the corresponding author up to 15 years after the end of the study.

## Abbreviations

AUMC: Amsterdam University Medical Centres
BMI: Body mass index
COVID-19: Coronavirus disease 2019
D0: Day 0 (baseline)
D7: Day 7
HIC: High-income country
HRQL: Health-related quality of life
ICU: Intensive care unit
LMIC: Low-/middle-income country
OECD: Organisation for Economic Co-operation and Development
RR: Respiratory rate
RT-PCR: Reverse transcriptase polymerase chain reaction
SARS-CoV-2: Severe acute respiratory syndrome coronavirus 2
SD: Standard deviation
SF-36: Medical Outcomes Study Short Form 36-item health survey
SpO_2_: Saturation of p*eripheral o*xygen
WHO: World Health Organization
