# Supplementary material for: Health-related quality of life among persons with initial mild, moderate, and severe or critical COVID-19 at 1 and 12 months after infection: a prospective cohort study
Source: BMC Med. 2022 Nov 2;20:422. doi: 10.1186/s12916-022-02615-7 (PMC9629769; doi:10.1186/s12916-022-02615-7)
Supplement: Supplementary file 1 — Additional file 1: Table S1. Comparison of demographic and clinical characteristics of RECoVERED study participants who did and did not complete at least 1 HRQL questionnaire. [file 12916_2022_2615_MOESM1_ESM.docx]

## Table S1. Socio-demographic, clinical (baseline and COVID-19-related) characteristics of RECoVERED study participants who did and did not complete at least 1 HRQL questionnaire

|  | **Participants who completed at least 1 HRQL questionnaire** | **Participants who did not complete at least 1 HRQL questionnaire** | **P-value** |
| --- | --- | --- | --- |
|  | N=269 | N=80 |  |
| Sex |  |  | 0.15 |
| Male | 145 (54%) | 51 (64%) |  |
| Female | 124 (46%) | 29 (36%) |  |
| Age (years), mean (SD) | 49 (16) | 52 (16) | 0.08 |
| BMI category |  |  | 0.31 |
| Normal weight | 113 (42%) | 26 (38%) |  |
| Overweight | 92 (35%) | 22 (31%) |  |
| Obese | 60 (23%) | 22 (31%) |  |
| Migration background |  |  | 0.03 |
| Dutch | 169 (64%) | 22 (48%) |  |
| OECD high-income | 35 (13%) | 5 (11%) |  |
| OECD low/middle income | 61 (23%) | 19 (41%) |  |
| Highest level of education |  |  | 0.04 |
| None, primary, secondary or vocational training | 100 (38%) | 22 (56%) |  |
| University education | 166 (62%) | 17 (44%) |  |
| Number of COVID-19 high-risk comorbidities |  |  | 0.23 |
| <3 | 249 (93%) | 70 (88%) |  |
| 3 or more | 20 (7%) | 10 (12%) |  |
| COVID-19 clinical severity |  |  | 0.006 |
| Mild | 82 (31%) | 17 (21%) |  |
| Moderate | 122 (45%) | 29 (36%) |  |
| Severe/critical | 65 (24%) | 34 (43%) |  |
| Hospital admission | 117 (44%) | 61 (76%) | <0.001 |
| ICU admission | 32 (12%) | 12 (15%) | 0.59 |

BMI=Body mass index; OECD= Organisation for Economic Co-operation and Development; HIC= high-income country; LMIC= low- or middle income country; ICU= Intensive Care Unit;

Continuous variables presented as mean (standard deviation) and compared using analysis of variance; categorical and binary variables presented as n(%) and compared using the Pearson χ2 test (or Fisher exact test if n<5).

Clinical severity groups defined as: mild as having a RR<20/min and SpO2 on room air >94% at both day 0 and 7; moderate disease as having a RR 20-30/min, SpO2 90-94% and/or receiving oxygen therapy at day 0 or 7; severe disease as having a RR>30/min or SpO2 < 90% at day 0 or 7; critical disease as requiring ICU admission.

COVID-related comorbidities are based on WHO Clinical Management Guidelines and include: cardiovascular disease (including hypertension), chronic pulmonary disease (excluding asthma), renal disease, liver disease, cancer, immunosuppression (excluding HIV, including previous organ transplantation), previous psychiatric illness and dementia.
